# Does Pelleted Starter Feed Restriction and Provision of Total Mixed Ration Ad Libitum during Weaning Influence the Behavior of Dairy Calves?

**DOI:** 10.3390/ani14192759

**Published:** 2024-09-24

**Authors:** Anna Antonella Spina, Marina Tortadès, Domenico Britti, Raffaella Grande, Valeria Maria Morittu

**Affiliations:** 1 Department of Medical and Surgical Sciences, Magna Græcia University of Catanzaro, 88100 Catanzaro, Italy; 2 Health and Happiness Group, H&H Research, Teagasc Moorepark, P61 K202 Fermoy, Ireland; marina.tortades@hh.global; 3 Department of Health Sciences, Magna Græcia University of Catanzaro, 88100 Catanzaro, Italy; britti@unicz.it (D.B.); raffaella.grande@studenti.unicz.it (R.G.); 4 Interdepartmental Services Centre of Veterinary for Human and Animal Health (CISVetSUA), Magna Græcia University of Catanzaro, 88100 Catanzaro, Italy

**Keywords:** animal welfare, dairy calves, calf weaning, resting time, sucking time, restricted feed intake, monitoring systems, precision farming

## Abstract

**Simple Summary:**

Milk removal, more commonly known as weaning, is considered a major stressor in the early life of a calf. If improperly managed, it can negatively impact both the calf’s performance and welfare. One of the most delicate aspects to address during a calf’s growth is nutrition, both liquid and solid, so that the calf can develop the rumen compartment correctly without precluding natural behavior and, at the same time, preserving business costs. The weaning calf, in addition to milk, generally receives calf starters, a very palatable solid feed rich in concentrates, but no source of roughage, although several studies emphasize the importance of fibrous feeds even at this stage of the animal’s life. The aim of this study was to evaluate the impact on the behavior and welfare of weaning calves resulting from the provision of Total Mixed Ration (TMR) as a forage source in addition to calf starter administered ad libitum or in restricted quantities. TMR is a diet widely used in dairy cow breeding, obtained by chopping and mixing all types of feeds, i.e., forages, concentrates and other ingredients, in order to achieve a homogeneous ration composition. The results obtained from this study indicate that the administration of TMR without restrictions of starter allows the young calf to better express its natural behavior, suggesting a better state of well-being.

**Abstract:**

Currently, in dairy farming, there is growing concern for the welfare of calves during the critical period between the separation from their dams and weaning. During weaning, rationed feeding is a practice used to improve feed efficiency and control the calves’ growth, but it could also have negative consequences associated with hunger and feed restriction behavior. One such consequence could be the performance by calves of stereotyped behaviors indicative of poor welfare, such as non-nutritive oral behaviors. We hypothesized that making a Total Mixed Ration (TMR) available to calves, in addition to the standard pelleted starter diet, thanks to its structural and nutritional characteristics, could help to focus the oral behavior of the subjects towards nutritional activities and therefore limit the development of stereotyped behaviors, even when the amount of starter is restricted. To test this hypothesis, 30 female Holstein calves (equipped with an accelerometer based on an ear tag), were randomly assigned to one of three treatments: a control diet (CTR) with an ad libitum calf starter but without TMR; Treatment 1 (TRT1) with both ad libitum calf starter and ad libitum TMR; Treatment 2 (TRT2) with ad libitum TMR and a restricted amount of a calf starter (50% of the intake starter of the control group day by day). All animals were kept in individual cages equipped with a slow-flow teat bucket apparatus for milk feeding and with access to separated buckets (one for drinking water, one for the starter, and one for the TMR) placed on the outside of the front gate of each cage. Sucking behavior, as well as resting, ruminating and activity behavior, was recorded individually from 7 days of life to weaning (63 d of age) by an automated monitoring system based on ear-tag accelerometers (SCR eSense, Allflex, Irving, TX, USA). The results showed that in the CTR group, there was a greater sucking activity compared to the TRT1 group (26.25 min/head/day vs. 16.83 min/head/day, *p* = 0.0181), while the TRT2 group showed intermediate values (20.41 min/head/day). We hypothesized that this increased sucking activity may be explained by the oral activity directed at sucking the Milk Bar tube observed only in CTR calves after complete consumption of available milk and could indicate a higher stress level than in the TRT1 group. The time spent resting was significantly lower for the CTR group compared to the TRT1 group (9.20 h/day vs. 11.15 h/day, *p* = 0.0049) while the TRT2 group was in an intermediate situation (10.65 h/day). Furthermore, the increase in time dedicated to rest observed in TRT1, in light of the good vitality of the calves shown by the daily patterns of resting behavior and daily activity, also seems to suggest an improvement in welfare conditions compared to calves receiving pelleted starter alone. Based on these results, we can deduce that providing TMR ad libitum in the diet of weaning calves could be an effective strategy to improve calf welfare due to the reduction in stereotyped oral activity. However, further studies are needed to establish threshold values useful for distinguishing physiological from pathological resting times in weaning calves raised in individual cages.

## 1. Introduction

Feeding management during the pre-weaning phase of dairy heifer calves is crucial for farm economics as it can impact the lifelong productivity of the animal [1].

During this period, young ruminants undergo several morphological and physiological changes in the rumen to be able to efficiently transform low-quality forage into high-quality protein through microbial fermentation [2,3,4].

One well-known strategy to stimulate rumen epithelium and microbiota development is to promote volatile fatty acid production through feeding concentrate feed rich in easily fermentable carbohydrates [4,5,6]. However, these solid diets could lead to a low rumen pH, having negative implications ranging from undesired microbial population shifts and inefficient feed digestion to metabolic diseases that can jeopardize the calves’ future performance [7,8].

More recently, several authors have encouraged the inclusion of forage in the diet during the pre-weaning period to increase rumen pH since dietary fiber stimulates saliva production, which acts as a natural buffer [9,10]. Also, supplementing with dietary forage could enhance rumen wall morphology, as when neutral detergent fiber (NDF) content was increased in calf feed, it enhanced the thickness of the muscle and mucosa layers while reducing rumen plaques [11,12].

The response of calves to stressors such as weaning has traditionally been assessed using not only growth parameters but also behavioral parameters [13] such as non-nutritive sucking, resting and rumination activity [14].

In fact, during artificial weaning, the mode of milk transfer does not always satisfy the calf’s motivation to suck. Thus, stereotypical behaviors such as excessive non-nutritive sucking on other calves’ body parts (cross-sucking) and on equipment and parts of the barn in which they are reared often occur [15].

However, non-nutritive sucking among calves or redirected to objects is a common behavior in pre-weaned calves [16], but it can occasionally persist in older animals, creating health and management problems [14,16]. For example, continued suckling behavior in heifers or cows is potentially associated with udder deformities, mastitis and milk loss [17].

One of the underlying causes of cross-sucking, and in general of abnormal behavior, could be insufficient milk or energy intake [18,19], and can be influenced by the weaning method and by a solid diet [19]. Indeed, it is known that hunger increases suckling motivation in calves [20]. This may be particularly important because, in most weaning programs, the calf is fed milk doses that are gradually reduced, despite the fact that the calf’s rumen is unable to digest solid feed sufficiently. Also influencing cross-sucking during weaning may be the frequency of milk intake and the type of artificial teat or bucket used [19].

Strategies like leaving the nipple bottle for 10–15 min to allow dry suckling or providing forage to satiate their appetite have been suggested to diminish cross-sucking [10,21] and stress in calves while increasing the development of rumination [22,23]. Nevertheless, the nature, quantity, and form of forage to be administered to young ruminants in order to improve their welfare and production have not been fully elucidated. We hypothesized that the presence of TMR ad libitum (even when the amount of starter is halved), with its different structural fiber, energy content, and palatability compared to a forage such as hay or silage, could help weaning calves limit stereotypic behaviors and, in particular, non-nutritive oral behaviors without compromising performance. Following the hypothesis, the objectives of this study were the following: (1) to evaluate the effect of the provision of TMR in weaning calves on non-nutritive sucking, resting and activity behavior; (2) to evaluate the different impact of the TMR provision in calves receiving ad libitum or restricted amounts of pelleted starter on the behavior of young cattle.

## 2. Materials and Methods

This study was conducted during the late autumn to spring (November 2020 to April 2021) at a commercial dairy farm located in Bisignano (CS), Calabria (South Italy). All procedures were approved by the Animal Ethic Committee of the Magna Graecia University of Catanzaro (protocol number: 545/2020).

### 2.1. Animal Management and Experimental Design

Thirty Holstein-Friesian dairy heifer calves (with a mean weight of 38.5 ± 1.96 kg at birth) were enrolled sequentially as they were born and remained on the study on pre-weaning behavior until they were completely weaned at 63 d of age. Within 2 h of birth, calves were housed individually in straw-bedding cages (93 × 205 cm, covering 1.91 m^2^) with visual and auditory contact between the calves.

A completely randomized design was used in which calves were randomly assigned to 1 of 3 feeding treatments: (1) an ad libitum calf pelleted starter (Control group, CTR; *n* = 10); (2) an ad libitum calf pelleted starter plus, from 21 d of age, an ad libitum TMR (Treated 1 group, TRT1; *n* = 10); and (3) a restricted amount of a calf pelleted starter (50% of the intake recorded in the CTR group day by day) plus, from 21 d of age, an ad libitum TMR (Treated 2 group, TRT2; *n* = 10).

**Figure 1:**
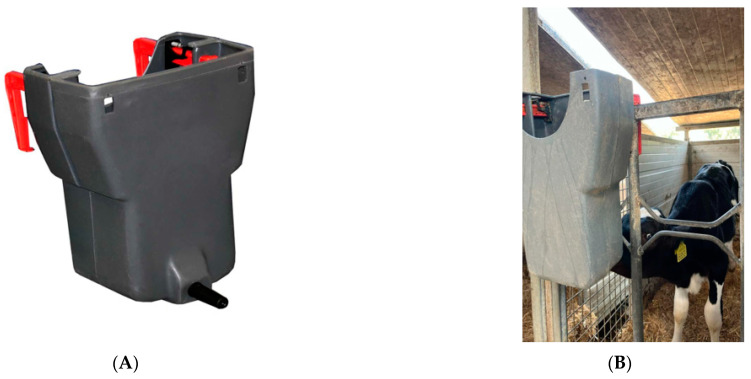
(**A**) Slow-flow teat bucket apparatus used to feed milk replacer to calves (Milk Bar 1 for calves w/EZ-Lock; Coburn Co., Whitewater, WI, USA) and (**B**) calf during the meal.

The diet composition and the milk administration schedule were that reported by Spina et al. [23] and are also included as Supplementary Material (Table S1). Briefly, a milk replacer (MR, ICIM spa, Mantova, Italy) was offered from the second meal to 56 d of age twice daily at 6:30 AM and 3:30 PM: 4 L/d from 2 to 7 d, 5 L/d from 8 to 14 d, 6 L/d from 15 to 20 d, and 8 L/d from 21 to 42 d of age. Then, the calves were stepped down to 6 L/d from 43 to 49 d and to 5 L/d from 50 to 56 d of age. From 57 to 63 d, calves were fed 2.5 L/d only once a day. MR was administered via a slow-flow teat bucket (Milk Bar 1 for calves w/EZ-Lock; Coburn Co., Whitewater, WI, USA, Figure 1) by 3 operators (10 calves per operator) who visually monitored the calves until the meal was completely ingested, waited and checked the ways and times the calves consumed milk and took care of removing the Milk Bar bucket for subsequent manual washing. If, within 40 min of feeding, any buckets were not completely emptied, the residual milk volume was measured and the calf was checked by the veterinarian. All calves had ad libitum access to water, to calf starter (for CTR and TRT1 groups) and to TMR (for TRT1 and TRT2 groups), distributed daily in separate buckets. The farm veterinarian monitored the health of the calves weekly, and researchers monitored the health and behavior of the calves daily.

### 2.2. Data Collection

For rumination, sucking, activity and resting data recording, within 3 d of age, calves were fitted with an ear tag activity sensor (SCR eSense, Allflex, Irving, TX, USA). The Allflex eSense™ ear tag sensor was positioned in the middle of the calf’s left ear, following the manufacturer’s instructions. As demonstrated in previous studies, the systems based on this technology appear to be valid for the reliable monitoring of daily rumination in cattle [22,23,24,25,26,27] and, recently, it was also used to measure the behavior of young livestock [28,29,30,31].

In short, this system recorded, through a 3D accelerometer, the angle and speed of ear and head movements. With these data, this system was able to determine the position of the neck and head and to distinguish between the different activities of the calves. Every 20 min, the data were transmitted from the ear tags to the system software that, through Allflex algorithms specially developed for weaning calves, reported them as sucking time, rumination, resting, activity and other unclassified behavior, expressed in minutes per 1 h intervals. The sum of these 5 parameters was always 60 min/h.

Data on sucking time (min/h), resting time (min/h), rumination time (min/h) and activity time (min/h), were processed to calculate the mean total daily time spent in the different conditions during the entire trial (7–62 d of age) and their daily pattern at each hour of the day (min/hour/head). Data recorded by the system before day 7 of age and on the day of weaning (day 63 of age) were not considered. In fact, the system needed 2–3 days to calibrate itself following ear tag application. On the other hand, on the last day of the weaning phase, the calf’s status changed from “on weaning” to “weaned”, resulting in a different algorithm being switched to correctly interpret the new behavioral pattern. A description of the behaviors observed and recorded by the automated Allflex system is summarized in Table 1.

### 2.3. Statistical Analyses

Statistical analyses were performed using Graph Pad PRISM software version 10.1.0 for Windows, La Jolla, CA, USA. The assumption of normality was evaluated by Shapiro–Wilk and Kolmogorov–Smirnov tests. Data on sucking activity (as well as activity, rumination and resting data), were analyzed using a linear mixed-effect model for repeated measures. The model included the effects of the diet “D” (CTR, TRT1, TRT2), time “T” (7 to 62 d of age of calves) and the interaction between diet and time “D × T” as fixed effects. The time was considered a repeated measure within the calf, and the calf was considered as a random effect. Tukey’s multiple comparison test was used to evaluate the difference between the averages. Sucking data (as well as resting, rumination and activity data), expressed as minutes per head/day, were processed to compare both daily sucking over the entire trial and sucking pattern over the 24-h day (min/hour/head) for the 3 groups under study. Again, the data were subjected to mixed-effects model analysis to evaluate the effect of the variables D and T and their interaction D × T. In particular, in the case of the interaction term, the null hypothesis was that any difference among diets is identical at all time-points. Results were expressed as expected marginal means (least squares means) ± SEM, and the significance level was established for *p* < 0.05, while the power (1-β) of the study was set at 80%.

## 3. Results

### 3.1. Calf Behavior

Data on the time (min/d) spent by the calves during the day on sucking, resting, ruminating and activity are shown in Table 2. Table 3, on the other hand, shows the daily patterns (min/h) related to sucking, resting, ruminating and activity of the three groups.

**Figure 2:**
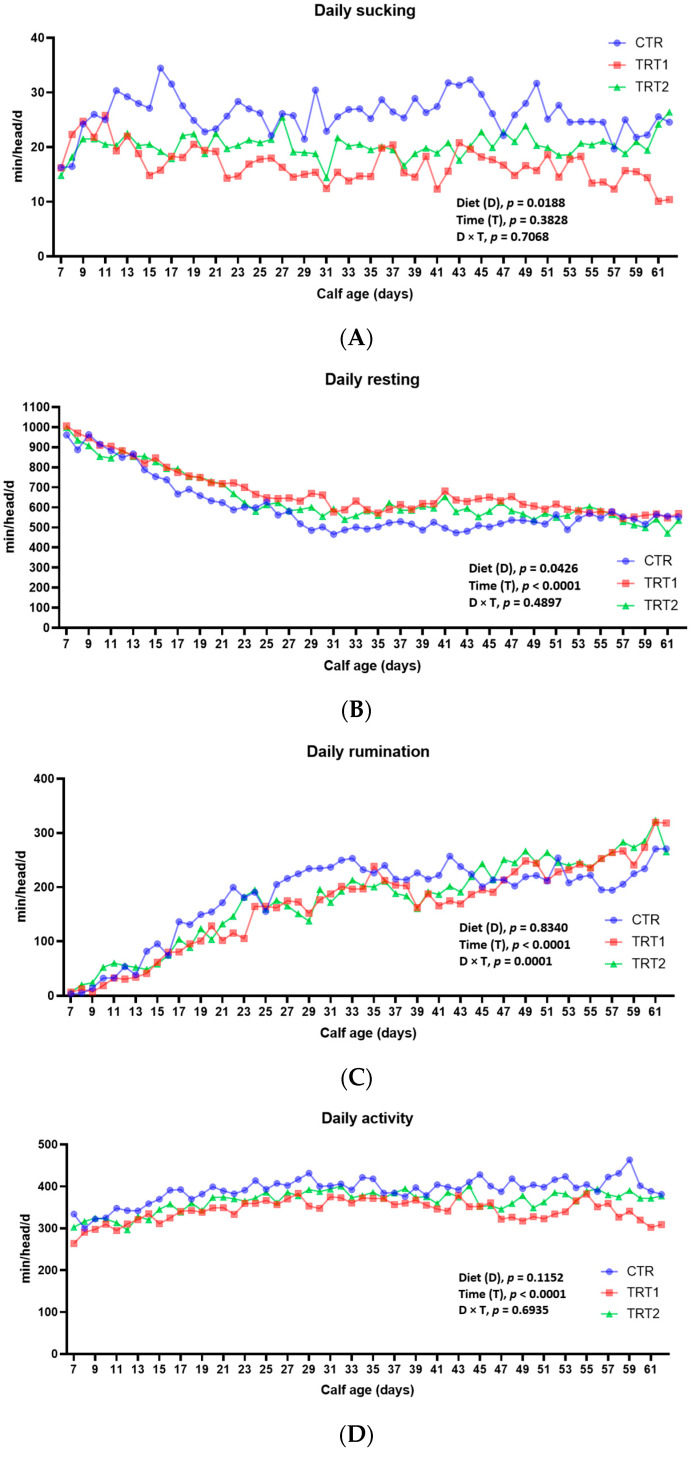
Daily duration of (**A**) sucking, (**B**) resting, (**C**) rumination and (**D**) activity behavior in weaning dairy calves from 7 d of age to the day before weaning according to the type of solid diet (CTR = only starter ad libitum; TRT1 = both starter and TMR ad libitum; TRT2 = restricted starter and TMR ad libitum). Each point on the graph indicates, according to the age of the subjects, the least squares mean of the total time (min/head/day) spent in the behavior considered.

Calves in the CTR group, which only received starter ad libitum, spent more time sucking compared to calves in the TRT1 group, which had been fed both starter and TMR ad libitum. More specifically, the CTR group spent almost 10 min more per day than the TRT1 group (*p* = 0.0188, Figure 2A) in sucking activities. Calves in the TRT2 group that received a rationed starter but ad libitum TMR were in an intermediate position between the other two groups; in fact, they demonstrated a sucking behavior not different from that of the CTR and TRT1 group. Regarding the time spent resting, the TRT1 group rested approximately 11 h per day, which was 1 h more than the CTR group, which spent almost 10 h per day at rest (*p* = 0.0426, Figure 2B). The TRT2 group, again, was in an intermediate position and statistically no different from the other two groups, as it rested approximately 10.5 h per day. Overall, throughout this study, minutes spent resting decreased significantly over time for all three groups (Time = *p* < 0.0001). For instance, at 7 days of life, the calves rested, on average, for 16 h a day. By 33 days, their daily rest decreased to almost 10 h, and finally, at 63 days of life, they reached 9 and a half hours of rest a day (Figure 2B).

Concerning time spent ruminating, as already reported in Spina et al. [20], this did not differ among feeding treatments (*p* = 0.8340, Figure 2C), but showed a significant effect of time (T, *p* < 0.0001), as well as a significant interaction of Diet × Time (D × T, *p* = 0.0001). In particular, the CTR group showed a more marked evolution of rumination until day 30, reaching a value of 235 min/d, and in the following days remaining close to this threshold. On the contrary, the treated groups showed a slower but more constant increase in rumination throughout the duration of the test, especially starting from 45 days of age, reaching values higher than the 235 min/d observed in the CTR group.

Regarding time spent on activities, we found no significant differences between groups (*p* = 0.1152), except for time (*p* < 0.0001), which led calves to be significantly more active during growth (Figure 2D). However, even if it does not reach statistical significance, from Figure 2D, one can observe the activities of the three groups throughout the duration of the test.

In fact, the calves in this group spent approximately 7 h a day active, compared to 6 h of activity in the TRT1 and TRT2 groups.

### 3.2. Calf Behavior Pattern

**Figure 3:**
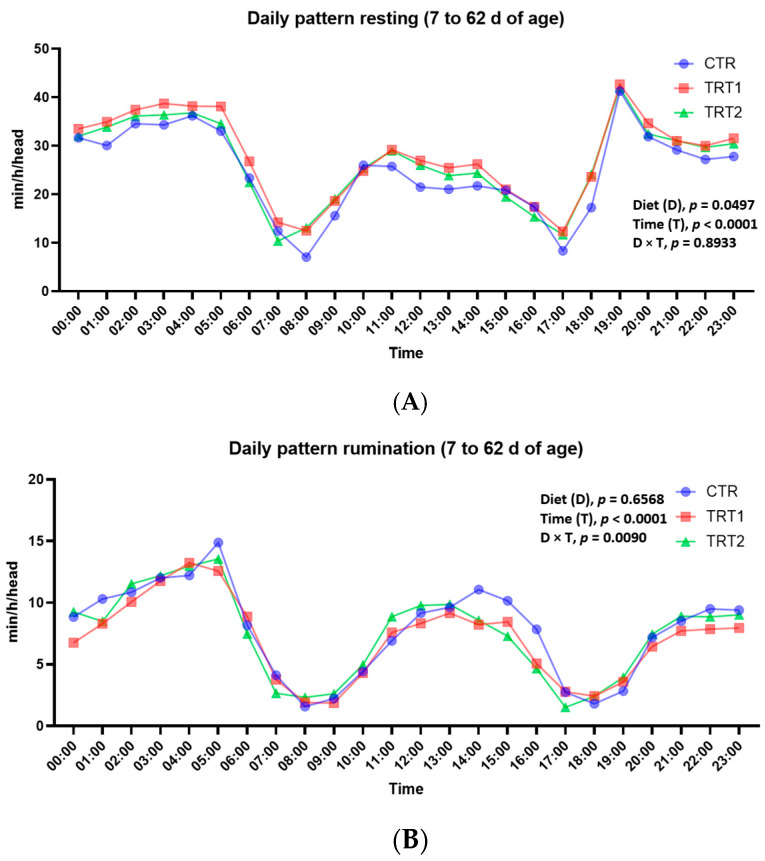

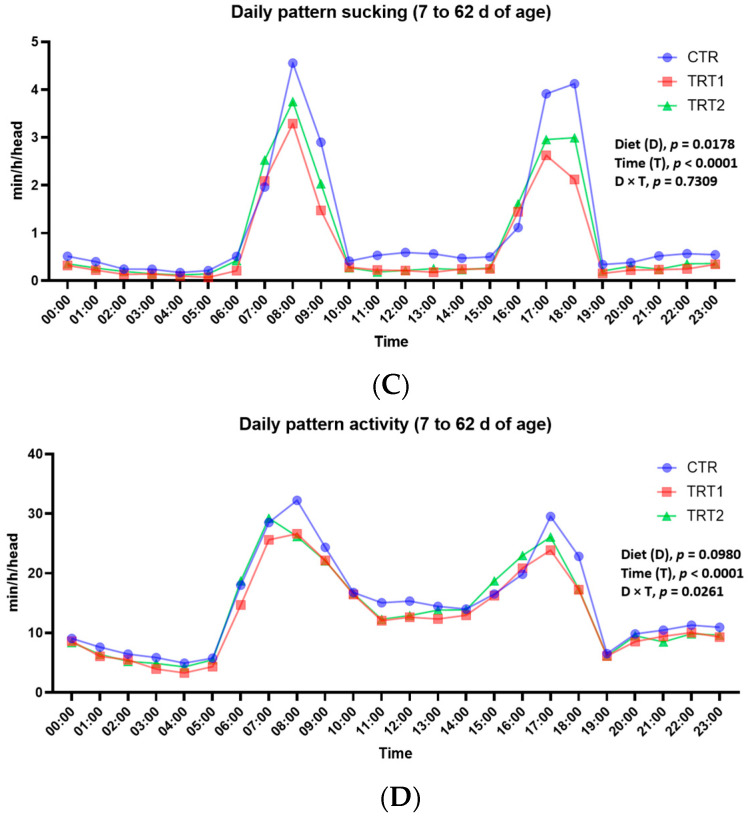
Daily patterns of (**A**) resting time, (**B**) rumination time, (**C**) sucking time and (**D**) activity time according to the type of solid diet (CTR = only starter ad libitum; TRT1 = both starter and TMR ad libitum; TRT2 = restricted starter and TMR ad libitum). The data points in the graphs indicate, for each hour of the day, the least squares mean calculated on the values (min/h/head) observed in calves from 7 days of age until the day before weaning.

As expected, the hours of greatest daytime rest were in the evening and at night, from 8:00 PM to 5:00 AM (Figure 3A). In similar time slots, calves also spent time ruminating, specifically between 10:00 AM and 4:00 PM (Figure 3B).

On the contrary, the time slots of greatest sucking (Figure 3C) and activity (Figure 3D) were in the morning and afternoon, especially those close to the time slots in which calves were fed. Specifically, morning peaks occurred between 6:00 AM and 9:00 AM, while in the afternoon they were between 4:00 PM and 6:00 PM (Figure 3A,D). Again, calves in the CTR group showed greater sucking activity than calves in the TRT1 group (*p* = 0.0178, Figure 3C) in the time slots close to mealtimes, while in the nighttime slots sucking activity was almost zero in all three groups. Finally, on the hourly activity patterns of calves, diet did not reach statistical significance (*p* = 0.0980). In fact, as can be seen from Figure 3D, in this case also, the CTR group shows a slightly higher activity pattern than the TRT1 group. The time of day significantly influenced the activities (*p* < 0.0001) since, as expected, peaks were observed in the daytime compared to the nighttime bands. The D × T interaction was also significant (*p* = 0.0261), indicating a greater activity of the CTR group, especially in the time bands close to milk-based meals, similarly to what was observed for sucking.

## 4. Discussion

In this study, we hypothesized that providing TMR ad libitum would help young calves focus their oral activities toward nutrients or solid feeds and to better express their natural behaviors, such as the need to ruminate and rest. We also hypothesized that introducing TMR ad libitum in diets with a significant starter restriction, due to its energy content and not only for the presence of structured fiber, could help to combat any starvation stress and, therefore, stereotyped behaviors.

The consumption of forage, such as hay, silage, and TMR, is considered a behavior enhancer in calves [32,33], and thus, the inclusion of a varied diet may be stimulating and preferred by young ruminants [34]. We also wanted to observe whether the TRT2 group, whose starter consumption had been reduced to 50% compared to the CTR group, showed negative performance and behavior compared to the other two groups.

Our results suggest different positive effects of TMR provision for the behavior of dairy calves. Interestingly, calves of the CTR (fed only starter ad libitum and without TMR) showed an increase in sucking compared with TRT1 (fed with TMR and starter ad libitum) but not compared with the TRT2 group (fed with starter intake restricted to 50% and TMR ad libitum). One possible explanation is that the calves of the TRT1 group, having both starter and TMR ad libitum, satisfied their hunger and had more opportunities to use their mouth in other feed-directed natural behaviors.

This aspect could suggest that the nature of the diet fed played a role in the expression of this behavior. Indeed, regardless of age, ruminants, if they are malnourished or deprived of forage, appear to show more abnormal behaviors that, although similar to the movements involved in feeding, are non-nutritive oral behaviors [35].

The time dedicated to non-nutritive sucking may be strictly related to the milk meal, where the calves, even if they have finished their milk, continue to suck, driven by the taste/odor of the milk and the presence of the teat. In this case, non-nutritive sucking was observed for a period ranging from 15 to 30 min per day. Therefore, it is not unusual to observe a calf performing non-nutritive sucking in proximity to milk meals, and within these time limits, it may not be worrying [36].

On the contrary, in the majority (about 70%) of cases, the most marked non-nutritive oral activities were observed away from milk meals. In our study, it was observed that the calves of the CTR group showed, compared to the TRT1 group, a greater sucking activity.

This phenomenon occurred in a manner strictly dependent on milk administration. In fact, after taking a few minutes to consume the volume of milk offered, the CTR calves continued to suckle the empty Milk Bar or, after its removal, the bar of the pen on which the Milk Bar had been placed.

Some authors suggest that oral behavior directed at the pen or empty teat could be related to the motivation to suck [18,37]. Therefore, in our case, we can assume that non-nutritive sucking is motivated by the taste and administration of milk, but in the CTR group, we must take into account that another small part of the non-nutritive sucking events was linked to other motivations, such as the need to have other feeds available of a different nature from the pelleted starter. Studies have already suggested that allowing calves to choose their own diet from a varied and balanced selection of ingredients is useful to satisfy some behavioral requirements [37,38].

Anyway, in our study, the recorded sucking might not necessarily be a cause for concern and an indication of abnormal behavior, since its duration was very short in all three groups. We recorded peaks of about 35 min per day of sucking, much less than those found by Pempek et al. [39], who recorded up to 47 min of sucking every 12 h.

However, we believe it is important to monitor this phenomenon, even if small in size; in fact, some evidence shows that not only calves that had a lot of non-nutritive sucking before weaning continued to show it once weaning was completed, but also many calves that had little non-nutritive sucking before weaning started to show this phenomenon once weaning was completed [36,40,41].

Interestingly, in the TRT1 group, apart from a lower sucking behavior, a greater resting time was also recorded. A good rest seems to be crucial for calf growth, as it preserves energy for the increases in and reconciles sleep with reflections on the regulation of the growth hormone. In fact, some studies have observed that calves that rested longer grew more [39,42,43].

As expected, calves in our study rested mainly at night. Feeding influenced the daily resting rhythm of calves. In fact, rest was interrupted around feeding, which was also reported by other studies [44,45] who noted, confirming our results, that rest started to decrease approximately 1.3 h before milk feeding.

The overall time spent at rest gradually decreases as the calves aged, as also previously observed in the literature [38,42]. A possible explanation is the reduced space in the pen as calves age, as confirmed by other authors [46] who have shown that resting positions require a lot of space, which, in the pen, decreases as the animal grows.

This less rest could also be explained by the greater time spent by older calves eating solid feed and ruminating. Indeed, the rumination and resting patterns we observed coincide, suggesting that young calves ruminate when resting and rarely when standing and engaged in other activities. This phenomenon is confirmed by Wang et al. [14], who argue that for dairy calves, standing rumination is rarely observed in behavioral observations, which indirectly indicates that the ruminant and resting behavior of dairy calves can be used as an indicator of their welfare [14].

Although the physiological functions of dairy calves during weaning require further investigation, based on the results observed in this study, we can suggest that the inclusion of forage via ad libitum TMR in the diet of young calves may help meet their behavioral and physiological needs and also may have a positive effect on post-weaning performance. However, the behaviors observed in this study, although they can be used as very useful attributes to indicate welfare, need to be supported by other factors that allow a more complete assessment of calf welfare during weaning and post-weaning.

## 5. Conclusions

In conclusion, starter restriction did not exacerbate sucking and other abnormal behaviors in dairy calves during weaning. Indeed, our results highlight the observed benefits of adding ad libitum TMR to the calf diet during this life stage, especially when added together with an unrestricted concentrate. However, although both the TRT1 and TRT2 groups had ad libitum TMR, only the TRT1 group, which also had ad libitum starter, seemed to cope better with the challenges of weaning in terms of behavior. This probably occurred because, based on their needs, calves in the TRT1 group had the possibility to choose between concentrate and TMR, both ad libitum.

Currently, studies on the behavior of calves during weaning carried out using the technology in this research appear to be few. We believe, however, that behavior-monitoring systems based on 3D accelerometers could constitute a valuable resource for future research on this topic.

In the future, further research should evaluate the effects of including TMR on the behavior of group-reared weaning calves, and, in particular, on the reduction in oral stereotypies both in the pre-weaning and subsequent phases.

## Figures and Tables

**Table 1:** Ethogram of behaviors observed during the 24 h of the day with ear tag sensors (SCR eSense, Allflex, Irving, TX, USA) in calves (7–62 d of age) housed in single pens.

Behavior	Description ^1^
Ruminating	Chewing preceded by regurgitation or followed by swallowing of a digestive bolus without intake of any feed (head up; ears toward the back).
Sucking	Calf is sucking or licking at objects, e.g., at the rubber teat of Milk Bar (with or without milk intake) or at the fence. Calf standing with head and neck directed upwards to take milk from the Milk Bar.
Resting	Lying on the chest or side with some head movement or with the head resting on the body or the ground, without ruminating.
Activity	The calf is standing and engaged in activities other than those described above.
Unclassified	Non-identifiable behavior from the automatic system.

^1^ Adapted according to the definitions of [24,25].

**Table 2:** Effect of diet (D), time, defined as days of calf age (T), and interaction (D × T) on calf behavior observed during weaning (day 7 to day 62 of age) and recorded by the Allflex automatic system.

Item	Diet				*p*-Value		
	CTR	TRT1	TRT2	SEM	Diet	Time	Diet × Time
Daily rumination (min/d)	183.2	166.2	176.2	4.58	0.8340	<0.0001	0.0001
Daily sucking (min/d)	26.1 ^a^	16.8 ^b^	20.3 ^ab^	0.58	0.0188	0.3828	0.7068
Daily resting (min/d)	601.0 ^b^	675.9 ^a^	645.9 ^ab^	18.07	0.0426	<0.0001	0.4897
Daily activity (min/d)	391.4	341.9	365.2	3.96	0.1152	<0.0001	0.6935

Average values (least squares mean) according to the type of solid diet (CTR = only starter ad libitum; TRT1 = both starter and TMR ad libitum; TRT2 = restricted starter and TMR ad libitum). SEM = standard error of the mean; values labeled with different lowercase letters within a row are different for *p* < 0.05.

**Table 3:** Effect of diet (D), time, defined as hour of the day (T), and interaction (D × T) on rumination, sucking, resting and activity of calves observed during weaning (day 7 to day 62 of age) and recorded by the Allflex automatic system.

Item	Diet				*p*-Value		
	CTR	TRT1	TRT2	SEM	Diet	Time	Diet × Time
Daily pattern rumination (min/h)	7.77	7.04	7.47	0.754	0.6568	<0.0001	0.0090
Daily pattern sucking (min/h)	1.10 ^a^	0.70 ^b^	0.85 ^ab^	0.274	0.0178	<0.0001	0.7309
Daily pattern resting (min/h)	24.81 ^b^	27.91 ^a^	26.65 ^ab^	1.808	0.0497	<0.0001	0.8933
Daily pattern activity (min/h)	14.85	12.88	13.72	1.626	0.0980	<0.0001	0.0261

Average values (least squares mean) according to the type of solid diet (CTR = only starter ad libitum; TRT1 = both starter and TMR ad libitum; TRT2 = restricted starter and TMR ad libitum). SEM = standard error of the mean; values labeled with different lowercase letters within a row are different for *p* < 0.05.

## Data Availability

Data supporting the findings of this study are available to anyone from the corresponding author upon reasonable request.

## Supplementary Materials

The following supporting information can be downloaded at https://www.mdpi.com/article/10.3390/ani14192759/s1, Table S1: Chemical composition and particle size distribution of Milk Replacer, calf starter and TMR.
