# Supplementary material for: Does Pelleted Starter Feed Restriction and Provision of Total Mixed Ration Ad Libitum during Weaning Influence the Behavior of Dairy Calves?
Source: Animals (Basel). 2024 Sep 24;14(19):2759. doi: 10.3390/ani14192759 (PMC11475125; doi:10.3390/ani14192759)
Supplement: Supplementary file 1 [file animals-14-02759-s001.zip › animals-3170697-supplementary.pdf]

Table S1. Chemical composition and particle size distribution of Milk Replacer, Calf Starter and TMR.

Chemical Composition	Milk Replacer [1]	Calf Starter [2]	TMR
Dry Matter (DM) %	11.42	88.5	55.55
Crude Protein % of DM	22.45	19.21	17.38
Ether Extract % of DM	20.19	4.29	2.63
Ash % of DM	7.12	8.02	7.89
Starch % of DM	-	33.33	13.15
NDF % of DM	-	21.45	48.26
UFL [3]/kg	1.10	1.05	0.73
**Particle size distribution [4], %**			
Long particles	-	-	8.94
Medium particles	-	-	39.32
Short particles	-	-	14.09
Fine particles	-	-	37.15

[1] Contained: Ca 1,13%; P 0.68%; Na 0.57%; lysine 2,15%.

[2] Contained, for kg, 25,160 IU of vitamin A, 4390 IU of vitamin D3, 62.8 mg of vitamin E, 24 mg of vitamin B1, 1,6 mg of vitamin B2, 1,0 mg of vitamin B6, 0.0114 mg of vitamin B12, 8.0 mg of niacinamide, 16 mg of calcium iodide, 36.96 mg of manganese oxide, 38,63 mg of zinc oxide and 0.5 mg of sodium.

[3] Net energy content calculated according to INRA 2018 standard for ruminants.

[4] Particle size was determined by a Penn State particle separator, which separates the particles into 4 fractions on 4 sieves: long (>19 mm), medium (8-19 mm), short (4-8 mm), and fine (< 4 mm).

The Calf Starter (BOVI-starter Pellet, PROGEO S.c.a., Reggio Emilia, Italy) was formulated with the following ingredients (descending percentage): corn, soybean meal, soybeans hulls, wheat bran, wheat middling, sugarcane molasses, corncob, sugar beet pulp, rice hull, calcium carbonate, calcium phosphate, soybean oil, sodium chloride.
The percentage of each ingredient is unknown as the calf starter was purchased from a licensed commercial feed mill.

The Total Mixed Ration (TMR) was mainly composed of (% DM): grass silage (20.55) and corn silage (12.20), wheat straw (25.65), sunflower extract meal (12.32), cornmeal (9.12) and calf starter (20.17).
The TMR was prepared on the same farm as the study and was balanced by a professional animal nutritionist.
